# The effect of sodium-glucose co-transporter-2 (SGLT2) inhibitors on blood interleukin-6 concentration: a systematic review and meta-analysis of randomized controlled trials

**DOI:** 10.1186/s12902-023-01512-1

**Published:** 2023-11-24

**Authors:** Sepehr Gohari, Faramarz Ismail-Beigi, Mahsa Mahjani, Saeed Ghobadi, Alireza Jafari, Hassan Ahangar, Sheida Gohari

**Affiliations:** 1 Student Research Center, School of Medicine, Zanjan University of Medical Sciences, Zanjan, Iran; 2 Department of Medicine, Case Western Reserve University, University Hospitals Cleveland Medical Center, Cleveland, OH USA; 3 Endocrine Research Center, School of Medicine, Shahid Beheshti University of Medical Sciences, Tehran, Iran; 4 Institute for Physical Activity and Nutrition (IPAN), School of Exercise and Nutrition Sciences, Deakin University, Melbourne, VIC Australia; 5 Physiology Research Center, Institute of Neuropharmacology, Kerman University of Medical Sciences, Kerman, Iran; 6 Department of Cardiology, School of Medicine, Mousavi Hospital, Zanjan University of Medical Sciences, Zanjan, Iran; 7 Department of Systems Science and Industrial Engineering, State University of New York at Binghamton, Binghamton, NY USA

**Keywords:** SGLT2 inhibitors, Interleukin-6, Diabetes mellitus, Inflammation, Cardiorenal protection, Randomized controlled trial, Meta-analysis

## Abstract

**Background:**

The low-grade chronic inflammation in diabetes plays an important role in development of cardiovascular and renal complications. Sodium-glucose co-transporter-2 (SGLT2) inhibitors are recognized as protective agents for cardio-renal complications. Interleukin-6 (IL-6) is positively associated with the pathophysiology of metabolic-related pathologies. The aim of this meta-analysis is to investigate the effect of SGLT2 inhibitors on blood IL-6 concentration in randomized controlled trials (RCTs).

**Methods:**

Embase, PubMed, and Scopus were systematically searched up to 1^st^ of November 2023. The eligible studies were RCTs with adult population that had provided blood IL-6 for both control and intervention groups. Cochrane risk-of-bias tool were for study quality assessment. Data were analyzed using random effect model via Stata statistical software.

**Results:**

Eighteen studies with a total of 5311 patients were included. Of which 3222 and 2052 patients were in intervention and control arm, respectively. Of the total population, 49.7% were men. The study durations ranged from 8 to 52 weeks. The pooled analysis showed a significant association between the use of SGLT2 inhibitors and lower IL-6 levels (standardized mean difference (SMD) = -1.04, Confidence Interval (CI): -1.48; -0.60, I^2^ = 96.93%). Dapagliflozin was observed to have a higher IL-6-lowering effect (SMD = -1.30, CI: -1.89; -0.71, I^2^ = 92.52) than empagliflozin or canagliflozin. Sub-group analysis of control groups (SMD = -0.58 (-1.01, -0.15) and -1.35 (-2.00, -0.70 for the placebo and active control sub-groups, respectively) and duration of interventions (SMD = -0.78 (-1.28, -0.28) and -1.20 (-1.86, -0.55) for study duration of ≤ 12 and > 12 weeks, respectively) did not change the results. Meta-regression analysis showed a significant correlation between the level of HbA_1c_ and IL-6-lowering efficacy of SGLT2 inhibitors.

**Conclusion:**

IL-6 levels are significantly reduced with the use of SGLT2 inhibitors with HbA_1c_ as the only marker influencing such reductions, and dapagliflozin had the highest potency. The anti-inflammatory effect of SGLT2 inhibitors supports their broader use to address diabetic complications related to inflammatory responses.

**Supplementary Information:**

The online version contains supplementary material available at 10.1186/s12902-023-01512-1.

## Introduction

The prevalence of diabetes, especially type 2 diabetes mellitus (T2DM), is increasing universally [1]. Hyperglycemia is believed to induce oxidative stress within tissues which triggers the formation of reactive oxygen species and cell death leading to enhanced cytokine infiltrations [2]. Circulatory interleukin-6 (IL-6) is commonly elevated in T2DM [3]. A growing body of evidence supports the critical role of IL-6 in the pathophysiology of cardiovascular and renal dysfunctions [4]. Genotyping for the IL-6 gene has revealed that IL-6 polymorphism is independently associated with coronary artery disease [5]. It has been established that IL-6 regulates glucose hemostasis, increased IL-6 levels might serve as an adaptive response to improve glycemic control [6]. However, IL-6 manifests a dual action against insulin resistance [7], while the cytokine enhances glucose uptake [8], serum IL-6 can predict the development of T2DM [9]. IL-6 as a downstream mediator of angiotensin II signaling can contribute to hypertensive disorders as well [10]. It is also one of the promoters of the Janus kinase/signal transducers and activators of transcription (JAK/STAT) signaling pathway [11]. Excessive amounts of IL-6 disturb the physiological balance of the cytokine’s signaling and leads to activation of JAK and STAT that can result in metabolic- and inflammatory -relate pathologies [12].

IL-6/ Soluble IL-6 receptor α (sIL-6Rα) pathway activate the pro-inflammatory trans-signaling in cells. More recently, blockade of this pathway in addition to neutralizing antibodies against IL-6 aiming at addressing the low-grade inflammation in patients with T2DM have shown beneficial effect [13, 14]. Sodium-glucose co-transporter-2 (SGLT2) inhibitors are glucose-lowering medications that have demonstrated protective effects against cardiorenal comorbidities [15]. Many studies have suggested that SGLT2 inhibitors exert promising anti-inflammatory characteristics [16]. Moreover, elevated IL-6 levels are strongly associated with poorly controlled diabetes. The effect of SGLT2 inhibitors on IL-6 levels has been assessed in several observational studies and in randomized controlled trials (RCTs); however, their overall impact on serum IL-6 has several discrepancies in the current literature. Therefore, in the present study we aimed to conduct a systematic review and meta-analysis of published RCTs to investigate the change in IL-6 levels with SGLT2 inhibitors.

## Materials and methods

The study was performed in accordance with PRISMA statement 2020 for the Preferred Reporting Items for Systematic Reviews and Meta-Analyses [17]. The protocol was prospectively registered in PROSPERO, https://www.crd.york.ac.uk/PROSPERO, ID: CRD42023393268.

### Literature search strategy

The authors systematically searched online databases including Embase, PubMed, and Scopus for relevant studies that were investigated until 1^st^ November 2023. The searches were conducted using the following Medline keywords: (Sodium-Glucose Transporter 2 Inhibitors and its drug names (gliflozins)) AND (Inflammation OR Interleukin-6 OR Tumor Necrosis Factors OR Cytokines OR Chemokines) AND (Randomized Controlled Trial and all the relevant keywords) with all their sub-trees in different combinations. The detailed search strategy is presented in supplementary file (Section A, Table S1). There was no restriction on language. Additionally, a manual search of references of related papers was performed to include any possible missed studies.

### Inclusion criteria

Two authors (SEP.G and M.M) had the responsibility of screening all searches to find eligible studies after removal of all duplicate reports. Afterwards, the eligible studies were classified based on their findings and data. Further, authors (SEP.G and SA.G) independently inspected all the screened papers. The PICO model that we used to s defined in supplementary file (Section B, Table S1). Only studies that met the following criteria were included in our meta-analysis: 1) Randomized controlled trial studies, 2) Human populations aged 18-year-old and above, 3) Studies that provided blood IL-6 for each group, and 4) Studies that compared SGLT2 inhibitors as the intervention group with a control group that used other glycemia-lowering agents, other medications, or placebo.

The exclusion criteria comprised of 1) participants with specific diseases that potentially carries alterations in serum IL-6 (e.g., malignancy, severe renal and liver failure), and if 2) the intervention period was less than 4 weeks.

### Data extraction

Data extraction was conducted according to a prepared checklist that consists of the following data: name of the first author, country of origin, year of publication, study design, study duration, type of intervention, study population, demographics, blood IL-6 and the related data for further analyses were extracted by two authors (SEP.G, M.M). In case the required data for the analyses was not reported in the main text, they were extracted from the figures by means of he online web application (https://apps.automeris.io/wpd/). Moreover, if the needed data hasn’t been mentioned, a request email was sent to the corresponding author obtain the data.

### Quality assessment and risk of bias

Cochrane risk-of-bias tool for randomized trials (RoB-2) [18] was used to methodologically assess the quality of evidence in each article by two independent authors (SA.G and M.M) with disagreements being determined by a third author (A.J, Sh.G). The domains of this scale are listed as randomization process, deviation from intended intervention, missing outcome data, measurement of the outcome, and the selection of the reported result. RoB-2 categorizes studies into low, high, and some concerns regarding the risk of bias. Finally, a traffic light plot was generated using Rob-2 excel assessment tool.

### Statistical analysis

Data was expressed as standardized mean differences with 95% confidence intervals (CI) using the generic inverse-variance method and random effects restricted maximum likelihood model. Whenever standard deviation (SD) change was not reported, we used [(SD baseline^2^ + SD final^2^)—(2 × R × SD baseline × SD final)]. We performed four meta-regression analyses to evaluate age, level of HbA_1c_ and sex effects on outcomes. Funnel plot and Egger's regression tests were used to assess publication bias. Statistical heterogeneity between studies was investigated using Higgins I^2^ statistics (> 50%), τ^2^, and the Cochrane Q test (*P* < 0.1) [19]. A statistical significance was considered Two-tailed *ρ* value < 0.05. All analyses were fulfilled by using Stata (Stata Statistical Software: Release V.15. College Station, Texas, USA: StataCorp LLC) [20].

## Results

### Study selection and characteristics

**Fig. 1:**
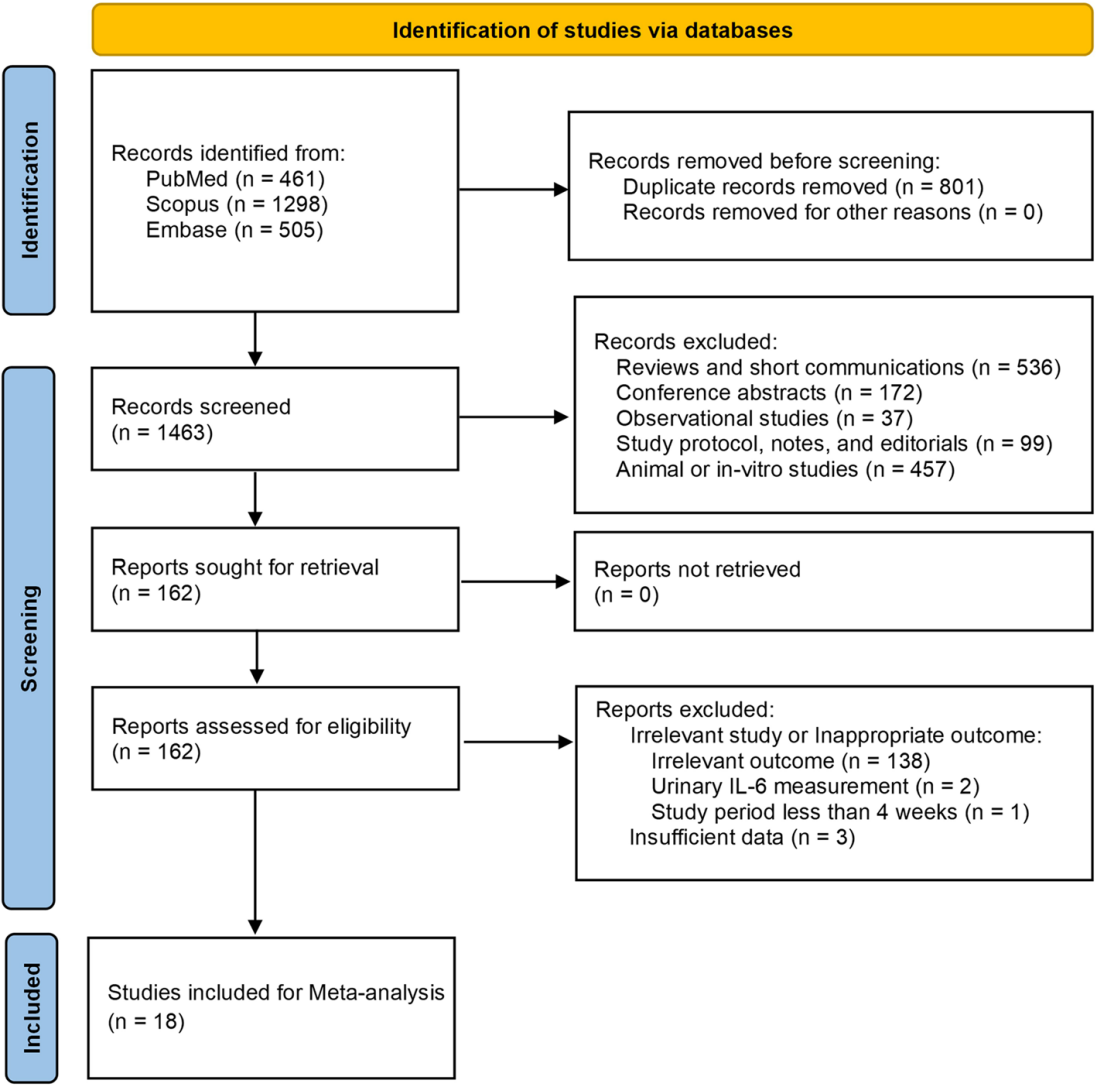
PRISMA flow-diagram of the included studies

The systematic search yielded 2264 potentially relevant records of which 801 were duplicates. Through screening the abstracts, 162 articles were assessed for full-text analysis. Among which, articles that reported irrelevant outcomes, urinary IL-6, had study periods less than 4 weeks, and had insufficient data were excluded. Following the exclusions, 18 articles were identified that met the inclusion criteria (Fig. 1). The eligible studies were published from 2018 to end of 2023 and, 3 were post-hoc analyses. The web application was used to extract the IL-6 data for 5 studies [21–25]. One study was in Chinese and was translated into English [26]. The complementary data for 1 study was obtained by email from the corresponding author [27].

The total number of patients included in the analysis was 5311. The median age was 49.5 years and 49.7% were male. Treatment durations varied from 8 to 52 weeks. Three studies had used canagliflozin [22, 24, 25], 9 had used dapagliflozin [21, 26, 28–34] and 6 had used empagliflozin [23, 27, 35–38]. With respect to active controls, 8 studies had compared with placebo and 10 had compared with other anti-glycemic agents (e.g., liraglutide, glibenclamide, metformin, sitagliptin, glimepiride) or another medication (valsartan and standard anti-heart failure drugs). Detailed information about the studies and their patients’ characteristics are presented in Table 1.

**Table 1:** Study and patients’ characteristics

*Author, year*	*Country*	*Treatment* *Duration*	*Intervention*		*Patients (n)*		*Age (years)*		*Male (%)*		*Baseline HbA1c (%)*	
			***Treatment***	***Control***	***Treatment***	***Control***	***Treatment***	***Control***	***Treatment***	***Control***	***Treatment***	***Control***
Shi [34] et al., 2023	China	24 weeks	Dapagliflozin 10 mg Once/Daily	Other Antidiabetic Drugs	40	38	49.0	47.4	27	27	8.38	8.66
Benedikt [33] et al., 2023	Austria	26 weeks	Empagliflozin 10 mg Once/Daily	Placebo Once/Daily	191	183	31.0	57.0	84	79	-	-
Song [38] et al., 2023	China	48 weeks	Dapagliflozin 5 mg Once/Daily	Standard Anti-heart failure Treatment	46	46	53.5	55.12	58.7	54.3	-	-
Ge [28] et al., 2022	China	24 weeks	Dapagliflozin 10 mg Once/Daily	Sacubitril /Valsartan 200 mg Twice/Daily	60	60	68.0	69.00	60	48.33	-	-
Zhang [32] et al., 2022	China	12 weeks	Dapagliflozin 10 mg Once/Daily	Placebo Once/Daily	28	28	57.68	56.25	71.43	92.86	-	-
Ou [21] et al., 2022	China	12 weeks	Liraglutide 0.6 mg + Dapagliflozin 5 mg Once/Daily	Liraglutide 0.6 mg Once/Daily	68	57	56.7	57.2	54.41	49.12	10.4	10.4
Koshino [22] et al., 2022	Netherland	48 weeks	Canagliflozin 100 + 300 mg Once/Daily	Placebo Once/Daily	2326	1177	62.9	62.5	67	67	8.2	8.2
Kobrynska [36] et al., 2022	Ukraine	24 weeks	Empagliflozin 10 mg Once/Daily	Metformin	51	49	-	-	-	-	-	-
Janić [33] et al., 2022	Slovenia	12 weeks	Empagliflozin 25 mg Once/Daily	Placebo Once/Daily	10	10	43.1	46.0	-	-	7.8	7.8
Huang [29] et al., 2022	China	12 weeks	Dapagliflozin 10 mg Once/Daily	Valsartan 80 mg Twice/Daily	60	60	56.21	55.67	56.7	60	9.31	9.31
Gohari [27] et al., 2022	Iran	26 weeks	Empagliflozin 10 mg Once/Daily	Placebo Once/Daily	47	48	62.08	63.6	48.9	33.3	8.05	7.75
Xue [31] et al., 2021	China	24 weeks	Dapagliflozin 10 mg Twice/Daily	Conventional anti-diabetic drugs	35	35	58.58	56.26	57.14	60	-	-
Sposito [37] et al., 2021	Brazil	12 weeks	Empagliflozin 10 mg Once/Daily	Glibenclamide 5 mg Once/Daily	48	49	57	58	60	61	7.9	7.9
Nandula [24] et al., 2021	USA	16 weeks	Canagliflozin 100 mg Once/Daily	Placebo Once/Daily	15	14	-	-	-	-	-	-
Bai [26] et al., 2021	China	12 weeks	Sitagliptin 100 mg + Dapagliflozin 10 mg Once/Daily	Sitagliptin 100 mg Once/Daily	40	40	48.61	45.61	50	55	-	-
Kahl [35] et al., 2020	Germany	24 weeks	Empagliflozin 25 mg Once/Daily	Placebo Once/Daily	42	42	62.7	61.5	69	69	6.8	6.7
Latva-Rasku [30] et al., 2019	Finland	8 weeks	Dapagliflozin 10 mg Once/Daily	Placebo Once/Daily	15	16	62	60	86.7	75	7	6.8
Garvey [25] et al., 2018	USA	52 weeks	Canagliflozin 300 mg Once/Daily	Glimepiride	100	100	58.5	57.5	48	55	7.8	7.7

### Meta-analysis results

**Fig. 2:**
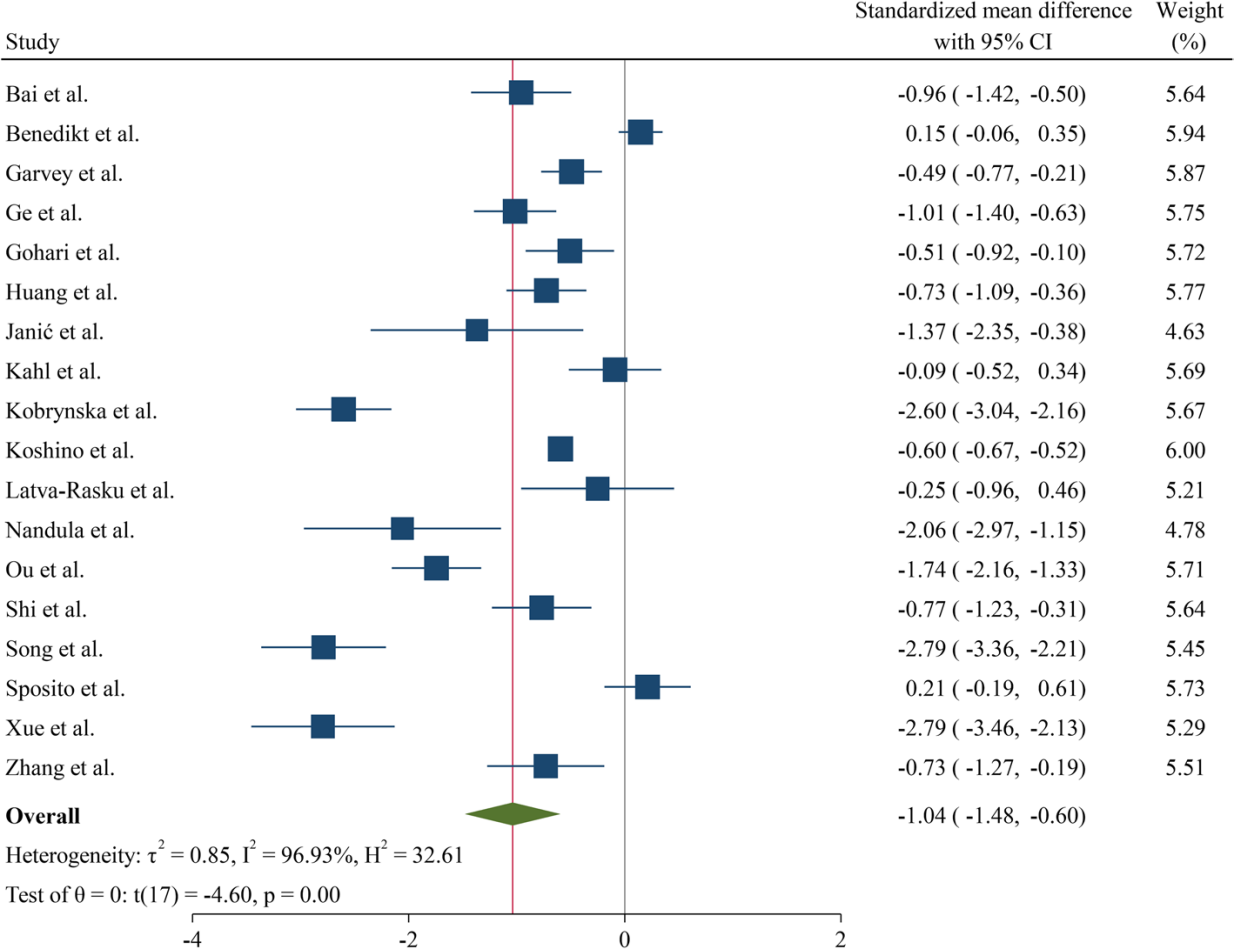
Forest plot of the effects of SGLT2 inhibitor drugs on blood interleukin-6. SGLT2 inhibitors: Sodium-glucose co-transporter-2 inhibitors

The pooled analysis of the 18 studies that were included showed a significant effect of SGLT2 inhibitors on blood IL-6 concentration, with a standardized mean difference (SMD) of (-1.04, CI: -1.48; -0.60). The intra-studies heterogeneity was significantly high (I^2^ = 96.93%) (Fig. 2).

**Fig. 3:**
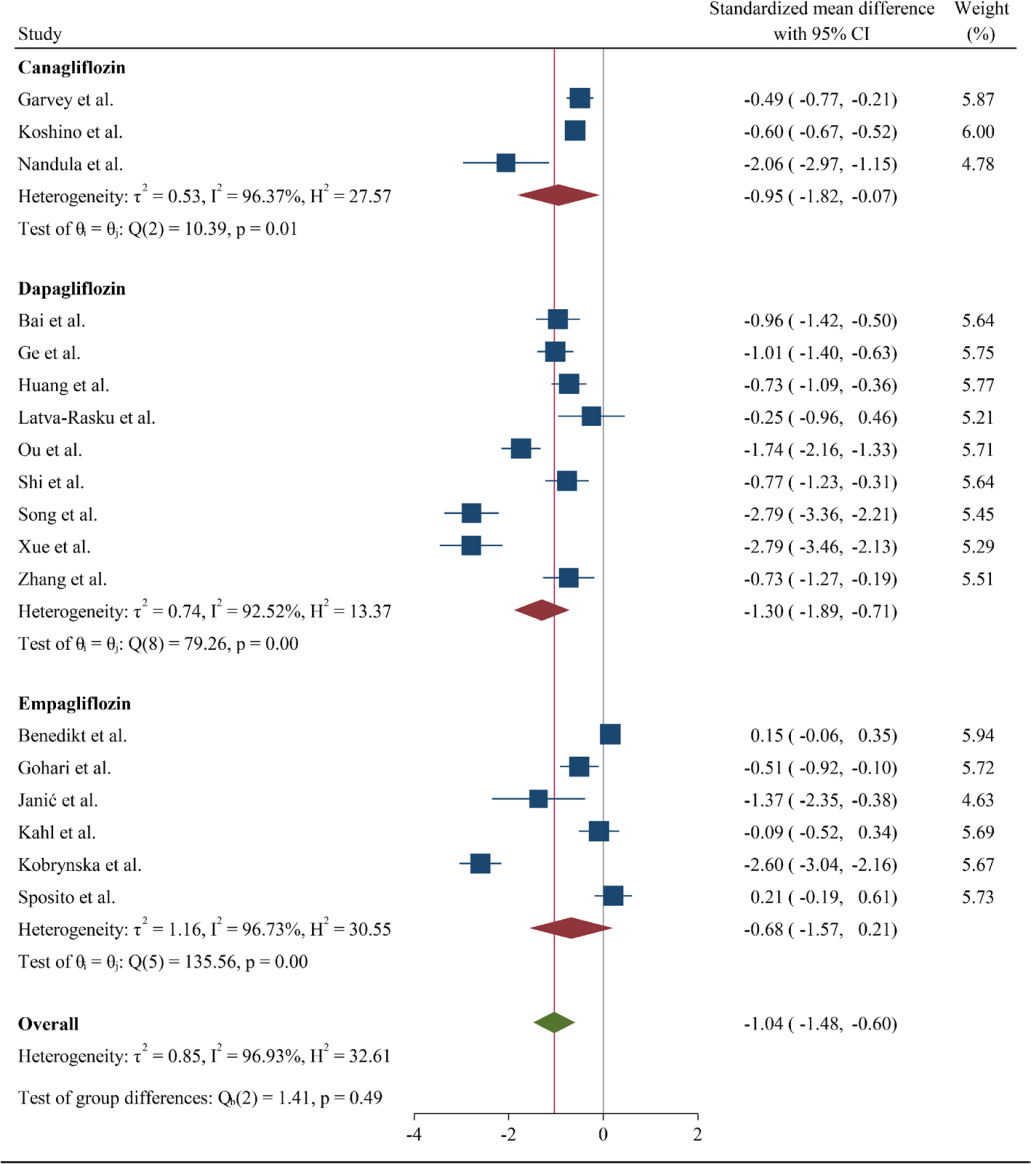
Forest plot summarizing the SMD of change scores among interleukin-6 between intervention and control arms for RCTs divided by the different types of gliflozins. SMD: Standardized mean difference. RCT: Randomized controlled trial

**Fig. 4:**
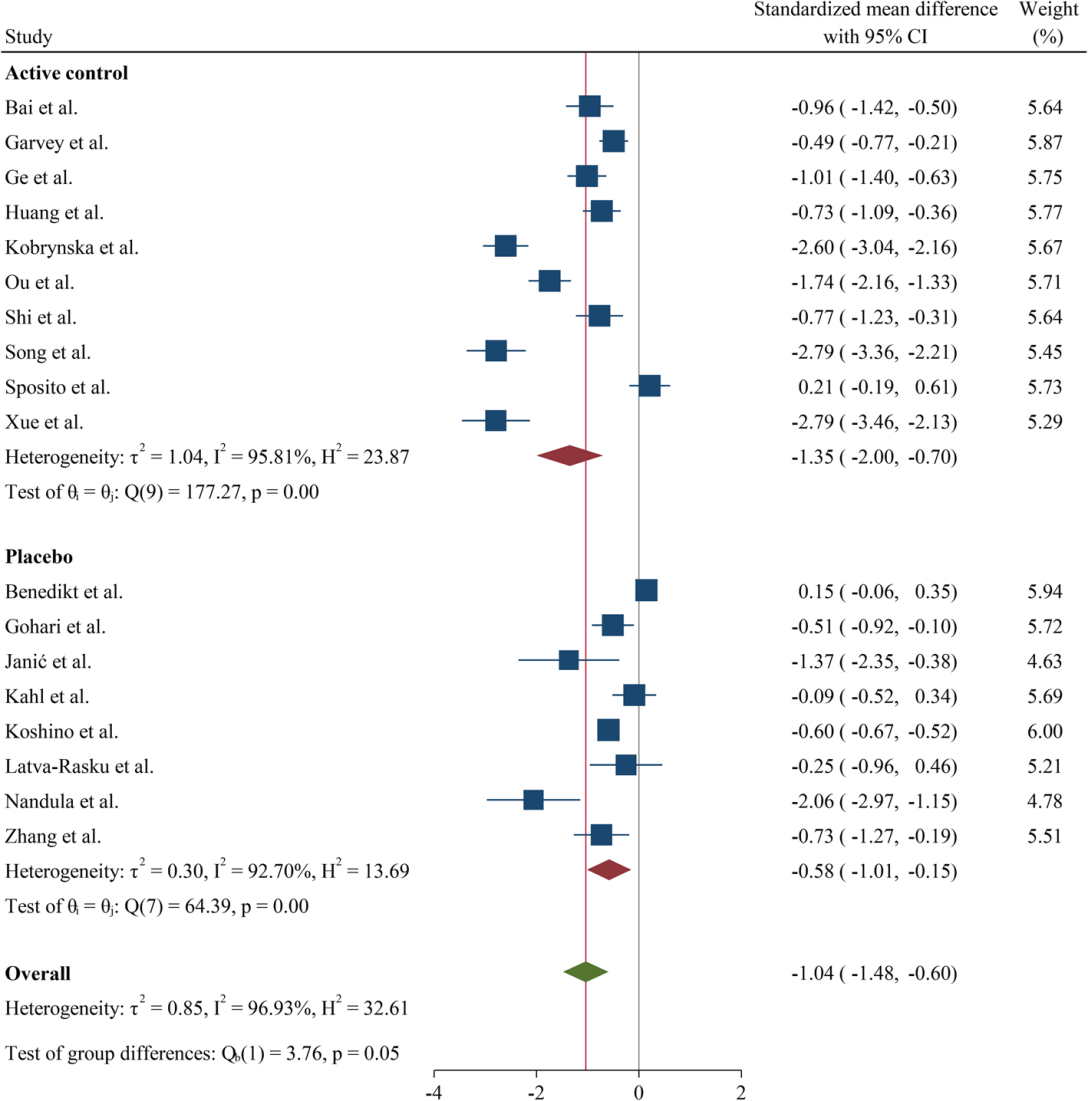
Forest plot summarizing the SMD of change scores among interleukin-6 between intervention and control arms for RCTs divided by either placebo or other medications (anti-diabetics or valsartan)

In sub-group analysis of the type of SGLT2 inhibitor employed, dapagliflozin was observed to have a relatively higher IL-6-lowering effect (SMD = -1.30, CI: -1.89; -0.71, I^2^ = 92.52%) compared to either canagliflozin or empagliflozin (Fig. 3). Sub-group analysis of control groups suggested no difference between use of placebo or other glycemia-lowering or other medications (SMD = -0.58 (-1.01, -0.15) and -1.35 (-2.00, -0.70) for the placebo and active control sub-groups, respectively) (Fig. 4).

The duration of intervention, either less or more than 12 weeks, also did not change the results (SMD = -0.78 (-1.28, -0.28) and -1.20 (-1.86, -0.55) for study duration of ≤ 12 and > 12 weeks, respectively) (Supplementary file, Section C, Figure S1). Additionally, the subgroup analysis of stratified HbA_1c_ levels indicated no significant difference (Supplementary file, Section C, Figure S2). The meta-regression analysis of age, gender and HbA_1C_ showed a significance correlation between IL-6-lowering efficacy of SGLT2 inhibitors with the level of HbA_1c_ at baseline (β = -0.403, 95% CI: -0.639, -0.166, *P* = 0.004) (Supplementary file, Section C, Table S1 and Figure S3). No correlations were found with the variables of age and male sex (Supplementary file, Section C, Figure S4 and Figure S5).

### Publication bias and sensitivity analyses

The randomization process was adequately generated in 5 (27.7%) trials; in the others the allocation was not concealed or not mentioned. In 6 (33.3%) studies, both patients and caregivers were blinded to the treatment and the judgment of the other trials declared “some concerns”. The assessor’s awareness of the intervention had disturbed the measurement of outcome as there were no information in 8 trials. In one study a pre-specific analysis plan for IL-6 was not finalized before reporting the results. The detailed risk of bias assessment is presented in supplementary file (Section D, Figure S1). The funnel plot showed no significant evidence of asymmetry (Supplementary file, Section D, Figure S2). Galbraith plot for heterogeneity analysis showed that there was no inconsistency across studies (Supplementary file, Section D, Figure S3). Leave-one-out and cumulative sensitivity analysis showed that the effect sizes were robust (Supplementary file, Section D, Figure S4). A sensitivity analysis was performed to assess the different correlation coefficients (r) for the main analysis. Neither different r nor using mean difference (MD) instead of SMD changed the result of the main analysis (Supplementary file, Section D, Table S1).

## Discussion

This meta-analysis showed that use of SGLT2 inhibitors is associated with a reduction in blood IL-6 levels. A prior meta-analysis was conducted on the effect of SGLT2 inhibitors on the biomarkers of inflammation; however, considering their inclusion of limited evidence in their assessment on IL-6, the study had failed to gain enough power to reach the significance level [39].

IL-6 exacerbates insulin resistance by activating STAT-3 in hepatocytes [40]. Current evidence has to some degree elucidated the mechanisms by which SGLT2 inhibitors regulate IL-6 levels [41, 42]. This class of medications suppress the mitochondrial complex I and inhibit intracellular glucose metabolism that leads to increased expression of AMPK signaling pathway and promote autophagy in immune cells; hence they exhibit anti-inflammatory effects [41–43]. Moreover, SGLT2 inhibitors hamper polarization of M2 macrophages, and as a result with decreased production of M1 macrophages, the release of IL-6 is moderated [44, 45]. Finally, it is worth emphasis that the effect of use of SGLT2 inhibitors on lowering IL-6 levels is not merely through control of glycemia because no reduction in IL-6 levels (compared to placebo) was found with use of other glycemia-lowering agents (Fig. 4).

IL-6 is considered to be an important contributor to kidney diseases [46]. Podocytes are great source of IL-6 secretion, and inevitably kidney is the first organ to be insulted by IL-6 [47]. This cytokine is involved in wide variety of glomerular and tubular pathological abnormalities [48]. In situ expression of IL-6 in diabetic nephropathy was significantly increased compared to a control group; hence higher IL-6 production may be associated with kidney injury in T2DM [49]. Furthermore, IL-6 in patients with T2DM is a robust trigger for the progression of chronic kidney disease [50], and high serum and urine IL-6 values have been proposed to be a prognostic marker for development of diabetic nephropathy [51].

Vascular cell aging via IL-6 signaling is hypothesized to accelerate atherosclerosis [52]. As IL-6 inhibition had markedly reduced biomarkers of thrombosis, IL-6 is thought to be a major contributing factor in myocardial ischemia and atherothrombotic complications [53, 54]. Furthermore, persistence elevated IL-6 concentrations can lead to cardiac hypertrophy mainly through IL-6 trans-signaling [55].

Reductions in circulating IL-6 have been shown to be associated with improved glycemic control in T2DM. Tocilizumab an anti-IL-6 receptor antibody decreased HbA_1c_ after 6 months of treatment [54]. Sarilumab, another IL-6 inhibitor, was associated with reduced HbA_1c_ level, seemingly independent of its anti-inflammatory effect [56]. Although IL-6 is known to be a key player in pancreatic beta-cell survival in diabetogenic conditions [57], IL-6 can couple autophagy to antioxidant response in beta-cells [58]. IL-6 was found to enhance insulin secretion in pancreatic islets and the associated hyperinsulinemia, suggesting that IL-6 plays a role in the pathogenesis insulin resistance [59, 60].

The disease-development properties of IL-6 have long been a topic of great interest. More recently, novel strategies are being implicated to target IL-6 for treatment of immune-mediated diseases through inhibition of the IL-6 signal transduction [61]. However, due to the duality of IL-6 function, blockade of IL-6 signaling has encountered some untoward complexities [62], and as such, interfering with the physiological homeostatic functions of IL-6 arguably should be avoided. In general, the therapeutic benefits must be weighed against the undesired effects of IL-6 blocking in T2DM.

In this meta-analysis, use of dapagliflozin was associated with a greater decrease in IL-6 levels compared to other medications in its own class, although the number of studies using canagliflozin was limited. The subgroup analysis of the control groups, use of SGLT2 inhibitors was more potent in lowering IL-6 than other glycemia-lowering agents. In addition, the duration of treatment with SGLT2 inhibitors, whether less or more than 12 weeks, did not change the results, however a relatively greater reduction in IL-6 was observed with longer duration of treatment. Meta-regression analysis showed that age and sex had no contributary effects in IL-6-lowering characteristic with SGLT2 inhibitors. Higher HbA_1c_ levels at baseline were found to be associated with more pronounced reductions in serum IL-6 according to our meta-regression analysis. It is noteworthy to mention that such lowering effect may not be merely related to the reduction of HbA1c because similar reduction in IL-6 concentrations was not found in the control arms of three included studies that compared empagliflozin with other glycemic-lowering agents in which decreased HbA1c were observed in both study groups [21, 27, 34]. However, the above-mentioned finding has been less assessed in the current literature and thus further studies are needed.

*Koshino *et al. observed that the increments of IL-6 with canagliflozin after one year had significantly lower slope than the controls and the differences was even wider by 6 years [22]. The limited upward slope of IL-6 levels with SGLT2 inhibitors advocates their anti-inflammatory role. Such effect of SGLT2 inhibitors on both IL-6 and glycemic level can uphold the use of this class of medications in T2DM. The potential mechanisms and clinical role of SGLT2 inhibitor on the complications of DM through regulating IL-6 is yet to be elucidated. Use of SGLT2 inhibitors to target inflammatory responses is not only focused on T2DM, but also in a wider range of diseases may warrant future large-scale studies.

This study has several strengths. First, this is the first meta-analysis that has gathered enough evidence regarding the changes in circulatory IL-6 with use of SGLT2 inhibitors. Second, our results appear to show that dapagliflozin is a more effective agent in this class of medications in attenuating IL-6 levels. Third, we have performed sensitivity analysis through reevaluating the data with three different correlation coefficients and all three represented the same results. Moreover, there were very narrow differences in between the SMDs and MDs with each coefficient. Altogether, such statistics have favored the results to achieve high reliability. Furthermore, it should be noted that although 40% of the included studies were from China, the rest of the studies were from different regions worldwide. Such diversity of the origin of the study population can highlight the generalizability of our results. We also acknowledge important limitations of our study. The main limitation was the marked heterogeneity across studies. Although, we have explored the source of heterogeneity through meta-regression and subgroup analysis, HbA_1c_ at baseline was the only factor identified to explain the heterogeneity.

## Conclusion

In this meta-analysis and systematic review, we have presented robust evidence that the pro-inflammatory biomarker, IL-6, is significantly reduced by SGLT2 inhibitors with HbA_1c_ as the only marker influencing such reduction. Use of dapagliflozin was associated with greater decrease in IL-6 levels compared to use of either empagliflozin or canagliflozin. The importance of these findings could be attributed to the cardiorenal protective properties of SGLT2 inhibitors. These findings arguably suggest that use of SGLT2 inhibitors could be considered in other inflammatory-related pathologies in patients with and without T2DM.

### Supplementary Information

**Additional file 1: Table S1. **We have searched PubMed, Embase, and Scopus databases using the terms below, PubMed for example (Updated to November 2023).** Table S1**. PICO inclusion criteria.** Table S1. **Meta-regression analysis of demographic and clinical variables on IL-6 lowering effect of SGLT2 inhibitors.** Figure S1. **Forest plot of the effect of SGLT2 inhibitor drugs on interleukin-6 based on the trial duration.** Figure S2. **Forest plot of the effect of SGLT2 inhibitor drugs on interleukin-6 based on the level of HbA1c.** Figure S3. **Relationship between level of HbA1c and interleukin-6 lowering effect of SGLT2 inhibitors. SGLT2 inhibitor: Sodium-glucose co-transporter-2 inhibitors.** Figure S4. **Relationship between male sex and interleukin-6 lowering effect of SGLT2 inhibitors. SGLT2 inhibitor: Sodium-glucose co-transporter-2 inhibitors.** Figure S5. **Relationship between age and interleukin-6 lowering effect of SGLT2 inhibitors. SGLT2 inhibitor: Sodium-glucose co-transporter-2 inhibitors.** Figure S1. **Risk of bias quality assessment results using ROB-2 tool.** Figure S2. **Funnel plot with pseudo 95% confidence limits demonstrating the SMD of interlukin-6 for each trial against their corresponding SEs. SMD: Standardized mean difference, SE: Standard error. Egger regression test had the *p*-value of 0.061.** Figure S3. **Galbraith plot to assess heterogeneity of the effects of SGLT2 inhibitor drugs on interleukin-6. SGLT2 inhibitor: Sodium-glucose co-transporter-2 inhibitors.** Figure S4. **The sensitivity analysis of the included studies using the leave-one-out (Left) and cumulative (Right) approaches.** Table S1. **Sensitivity analyses to assess the effect of different correlation coefficients on the main results.

## Data Availability

The datasets used and/or analyzed during the current study available from the corresponding author on reasonable request.

## Abbreviations

T2DM: Type 2 diabetes mellitus
IL-6: Interleukin 6
JAK/STAT pathway: Janus kinase/signal transducer and activator of transcription pathway
SGLT2 inhibitors: Sodium-glucose co-transporter-2 inhibitors
DM: Diabetes mellitus
RCT: Randomized controlled trial
SMD: Standardized mean difference
MD: Mean difference
AMPK pathway: AMP-activated protein kinase pathway
moAbs: Monoclonal antibodies

## References

[1] Sun H, Saeedi P, Karuranga S, Pinkepank M, Ogurtsova K, Duncan BB (2022). IDF diabetes atlas: Global, regional and country-level diabetes prevalence estimates for 2021 and projections for 2045. Diabetes Res Clin Pract.

[2] Oguntibeju OO (2019). Type 2 diabetes mellitus, oxidative stress and inflammation: examining the links. Int J Phys, Pathophysiol Pharmacol.

[3] Mirza S, Hossain M, Mathews C, Martinez P, Pino P, Gay JL (2012). Type 2-diabetes is associated with elevated levels of TNF-alpha, IL-6 and adiponectin and low levels of leptin in a population of Mexican Americans: a cross-sectional study. Cytokine.

[4] Kreiner FF, Kraaijenhof JM, von Herrath M, Hovingh GKK, von Scholten BJ (2022). Interleukin 6 in diabetes, chronic kidney disease, and cardiovascular disease: mechanisms and therapeutic perspectives. Expert Rev Clin Immunol.

[5] Rai H, Colleran R, Cassese S, Joner M, Kastrati A, Byrne RA (2021). Association of interleukin 6–174 G/C polymorphism with coronary artery disease and circulating IL-6 levels: a systematic review and meta-analysis. Inflamm Res.

[6] Lehrskov LL, Christensen RH. The role of interleukin-6 in glucose homeostasis and lipid metabolism. In Seminars in Immunopathology. Berlin/Heidelberg: Springer Berlin Heidelberg. 2019;41:491–99. 10.1007/s00281-019-00747-2 31101976

[7] Carey A, Febbraio M (2004). Interleukin-6 and insulin sensitivity: friend or foe?. Diabetologia.

[8] Stouthard J, Elferink RO, Sauerwein H (1996). Interleukin-6 enhances glucose transport in 3T3-L1 adipocytes. Biochem Biophys Res Commun.

[9] Kim JH, Bachmann RA, Chen J (2009). Interleukin-6 and insulin resistance. Vitam Horm.

[10] Zhang W, Wang W, Yu H, Zhang Y, Dai Y, Ning C (2012). Interleukin 6 underlies angiotensin II–induced hypertension and chronic renal damage. Hypertension.

[11] Aliyu M, Zohora FT, Anka AU, Ali K, Maleknia S, Saffarioun M (2022). Interleukin-6 cytokine: An overview of the immune regulation, immune dysregulation, and therapeutic approach. Int Immunopharmacol.

[12] Gurzov EN, Stanley WJ, Pappas EG, Thomas HE, Gough DJ (2016). The JAK/STAT pathway in obesity and diabetes. FEBS J.

[13] Kuryłowicz A, Koźniewski K (2020). Anti-inflammatory strategies targeting metaflammation in type 2 diabetes. Molecules.

[14] Moshapa FT, Riches-Suman K, Palmer TM (2019). Therapeutic targeting of the proinflammatory IL-6-JAK/STAT signalling pathways responsible for vascular restenosis in type 2 diabetes mellitus. Cardiol Res Pract.

[15] Bonnet F, Scheen A (2018). Effects of SGLT2 inhibitors on systemic and tissue low-grade inflammation: the potential contribution to diabetes complications and cardiovascular disease. Diabetes Metab.

[16] La Grotta R, de Candia P, Olivieri F, Matacchione G, Giuliani A, Rippo MR (2022). Anti-inflammatory effect of SGLT-2 inhibitors via uric acid and insulin. Cell Mol Life Sci.

[17] Page MJ, McKenzie JE, Bossuyt PM, Boutron I, Hoffmann TC, Mulrow CD (2021). The PRISMA 2020 statement: an updated guideline for reporting systematic reviews. Int J Surg.

[18] Sterne JA, Savović J, Page MJ, Elbers RG, Blencowe NS, Boutron I (2019). RoB 2: a revised tool for assessing risk of bias in randomised trials. bmj.

[19] Higgins JPT, Thomas J, Chandler J, Cumpston M, Li T, Page MJ, Welch VA (editors). Cochrane Handbook for Systematic Reviews of Interventions version 6.4 (updated August 2023). Cochrane. 2023. Available from www.training.cochrane.org/handbook.

[20] StataCorp L (2017). Stata statistical software: Release 15 (2017).

[21] Ou T, Wang W, Yong H, Hao H, Wang R, Dai X (2022). Liraglutide plus Dapagliflozin for high uric acid and Microalbuminuria in diabetes mellitus complicated with metabolic syndrome. Alter Ther Health Med.

[22] Koshino A, Schechter M, Sen T, Vart P, Neuen BL, Neal B (2022). Interleukin-6 and cardiovascular and kidney outcomes in patients with type 2 diabetes: new insights from CANVAS. Diabetes Care.

[23] Janić M, Cankar M, Šmid J, France Štiglic A, Jerin A, Šabovič M (2022). Empagliflozin-Metformin combination has antioxidative and anti-inflammatory properties that correlate with vascular protection in adults with type 1 diabetes. J Diabetes Res.

[24] Nandula SR, Kundu N, Awal HB, Brichacek B, Fakhri M, Aimalla N (2021). Role of Canagliflozin on function of CD34+ ve endothelial progenitor cells (EPC) in patients with type 2 diabetes. Cardiovasc Diabetol.

[25] Garvey WT, Van Gaal L, Leiter LA, Vijapurkar U, List J, Cuddihy R (2018). Effects of canagliflozin versus glimepiride on adipokines and inflammatory biomarkers in type 2 diabetes. Metabolism.

[26] Bai Xiaogang, Wang Jing, Bai Ting, Li Xia. Clinical study of dapagliflozin combined with sitagliptin in the treatment of brittle type 2 diabetes. Drug Eval Res. 2021;44(1):157–60.

[27] Gohari S, Reshadmanesh T, Khodabandehloo H, Karbalaee-Hasani A, Ahangar H, Arsang-Jang S (2022). The effect of EMPAgliflozin on markers of inflammation in patients with concomitant type 2 diabetes mellitus and coronary artery disease: the EMPA-CARD randomized controlled trial. Diabetol Metab Syndr.

[28] Ge T, Yang Y, Zhao Y. A study of the efficacy of sacubitril/valsartan plus dapagliflozin combination treatment in pulmonary arterial hypertension due to left heart disease. Perfusion. 2023;38(8):1697–704. 10.1177/02676591221127924 36173344

[29] Huang Y, Lu W, Lu H (2022). The clinical efficacy and safety of dapagliflozin in patients with diabetic nephropathy. Diabetol Metab Syndr.

[30] Latva-Rasku A, Honka M-J, Kullberg J, Mononen N, Lehtimäki T, Saltevo J (2019). The SGLT2 inhibitor dapagliflozin reduces liver fat but does not affect tissue insulin sensitivity: a randomized, double-blind, placebo-controlled study with 8-week treatment in type 2 diabetes patients. Diabetes Care.

[31] Xue L, Yuan X, Zhang S, Zhao X (2021). Investigating the effects of dapagliflozin on cardiac function, inflammatory response, and cardiovascular outcome in patients with STEMI complicated with T2DM after PCI. Evid-Based Complement Altern Med.

[32] Zhang H, Liu Z (2022). Effects of Dapagliflozin in combination with metoprolol sustained-release tablets on prognosis and cardiac function in patients with acute myocardial infarction after PCI. Comput Math Methods Med.

[33] Song B, Zhang H, Zhou B. Effects of Dapagliflozin on myocardial remodeling, inflammatory factors, and cardiac events in heart failure with preserved ejection fraction. Naunyn-Schmiedeberg's Archives of Pharmacology. 2023:1–10. 10.1007/s00210-023-02590-7. 10.1007/s00210-023-02590-7 37368031

[34] Shi M, Zhang H, Wang W, Zhang X, Liu J, Wang Q (2023). Effect of dapagliflozin on liver and pancreatic fat in patients with type 2 diabetes and non-alcoholic fatty liver disease. J Diabetes Complications.

[35] Kahl S, Gancheva S, Straßburger K, Herder C, Machann J, Katsuyama H (2020). Empagliflozin effectively lowers liver fat content in well-controlled type 2 diabetes: a randomized, double-blind, phase 4, placebo-controlled trial. Diabetes Care.

[36] Kobrynska O, Didushko O (2022). options of correcting insulin resistance and proinflammatory cytokine levels in patients with type 2 diabetes mellitus. Prob Endocrine Pathol.

[37] Sposito AC, Breder I, Soares AA, Kimura-Medorima ST, Munhoz DB, Cintra RM (2021). Dapagliflozin effect on endothelial dysfunction in diabetic patients with atherosclerotic disease: a randomized active-controlled trial. Cardiovasc Diabetol.

[38] Benedikt M, Mangge H, Aziz F, Curcic P, Pailer S, Herrmann M (2023). Impact of the SGLT2-inhibitor empagliflozin on inflammatory biomarkers after acute myocardial infarction–a post-hoc analysis of the EMMY trial. Cardiovasc Diabetol.

[39] Wang D, Liu J, Zhong L, Li S, Zhou L, Zhang Q (2022). The effect of sodium-glucose cotransporter 2 inhibitors on biomarkers of inflammation: A systematic review and meta-analysis of randomized controlled trials. Front Pharmacol.

[40] Senn JJ, Klover PJ, Nowak IA, Mooney RA (2002). Interleukin-6 induces cellular insulin resistance in hepatocytes. Diabetes.

[41] Fu J, Xu H, Wu F, Tu Q, Dong X, Xie H (2022). Empagliflozin inhibits macrophage inflammation through AMPK signaling pathway and plays an anti-atherosclerosis role. Int J Cardiol.

[42] Yang L, Liang B, Li J, Zhang X, Chen H, Sun J (2022). Dapagliflozin alleviates advanced glycation end product induced podocyte injury through AMPK/mTOR mediated autophagy pathway. Cell Signal.

[43] Xu C, Wang W, Zhong J, Lei F, Xu N, Zhang Y (2018). Canagliflozin exerts anti-inflammatory effects by inhibiting intracellular glucose metabolism and promoting autophagy in immune cells. Biochem Pharmacol.

[44] Lee T-M, Chang N-C, Lin S-Z (2017). Dapagliflozin, a selective SGLT2 Inhibitor, attenuated cardiac fibrosis by regulating the macrophage polarization via STAT3 signaling in infarcted rat hearts. Free Radical Biol Med.

[45] Xu L, Ota T (2018). Emerging roles of SGLT2 inhibitors in obesity and insulin resistance: Focus on fat browning and macrophage polarization. Adipocyte.

[46] Magno AL, Herat LY, Carnagarin R, Schlaich MP, Matthews VB (2019). Current knowledge of IL-6 cytokine family members in acute and chronic kidney disease. Biomedicines.

[47] Su H, Lei C-T, Zhang C (2017). Interleukin-6 signaling pathway and its role in kidney disease: an update. Front Immunol.

[48] Feigerlová E, Battaglia-Hsu S-F (2017). IL-6 signaling in diabetic nephropathy: From pathophysiology to therapeutic perspectives. Cytokine Growth Factor Rev.

[49] Araújo LS, Torquato BGS, da Silva CA, dos Reis Monteiro MLG, dos Santos Martins ALM, da Silva MV (2020). Renal expression of cytokines and chemokines in diabetic nephropathy. BMC Nephrol.

[50] Durlacher-Betzer K, Hassan A, Levi R, Axelrod J, Silver J, Naveh-Many T (2018). Interleukin-6 contributes to the increase in fibroblast growth factor 23 expression in acute and chronic kidney disease. Kidney Int.

[51] An Z, Qin J, Bo W, Li H, Jiang L, Li X (2022). Prognostic value of serum interleukin-6, NF-κB plus MCP-1 assay in patients with diabetic nephropathy. Dis Markers.

[52] Tyrrell DJ, Goldstein DR (2021). Ageing and atherosclerosis: vascular intrinsic and extrinsic factors and potential role of IL-6. Nat Rev Cardiol.

[53] Mascareno E, El-Shafei M, Maulik N, Sato M, Guo Y, Das DK (2001). JAK/STAT signaling is associated with cardiac dysfunction during ischemia and reperfusion. Circulation.

[54] Ridker PM, Devalaraja M, Baeres FM, Engelmann MD, Hovingh GK, Ivkovic M (2021). IL-6 inhibition with ziltivekimab in patients at high atherosclerotic risk (RESCUE): a double-blind, randomised, placebo-controlled, phase 2 trial. The Lancet.

[55] Booz GW, Day JN, Baker KM (2002). Interplay between the cardiac renin angiotensin system and JAK-STAT signaling: role in cardiac hypertrophy, ischemia/reperfusion dysfunction, and heart failure. J Mol Cell Cardiol.

[56] Genovese MC, Burmester GR, Hagino O, Thangavelu K, Iglesias-Rodriguez M, John GS (2020). Interleukin-6 receptor blockade or TNFα inhibition for reducing glycaemia in patients with RA and diabetes: post hoc analyses of three randomised, controlled trials. Arthritis Res Ther.

[57] Choi S-E, Choi K-M, Yoon I-H, Shin J-Y, Kim J-S, Park W-Y (2004). IL-6 protects pancreatic islet beta cells from pro-inflammatory cytokines-induced cell death and functional impairment in vitro and in vivo. Transpl Immunol.

[58] Marasco MR, Conteh AM, Reissaus CA, Cupit JE, Appleman EM, Mirmira RG (2018). Interleukin-6 reduces β-cell oxidative stress by linking autophagy with the antioxidant response. Diabetes.

[59] da Silva Krause M, Bittencourt A, de HomemBittencourt PI, McClenaghan NH, Flatt PR, Murphy C (2012). Physiological concentrations of interleukin-6 directly promote insulin secretion, signal transduction, nitric oxide release, and redox status in a clonal pancreatic-cell line and mouse islets. J Endocrinol.

[60] Rehman K, Akash MSH, Liaqat A, Kamal S, Qadir MI, Rasul A (2017). Role of interleukin-6 in development of insulin resistance and type 2 diabetes mellitus. Crit Rev™ Eukaryotic Gene Express.

[61] Ridker PM, Rane M (2021). Interleukin-6 signaling and anti-interleukin-6 therapeutics in cardiovascular disease. Circ Res.

[62] Garbers C, Heink S, Korn T, Rose-John S (2018). Interleukin-6: designing specific therapeutics for a complex cytokine. Nat Rev Drug Discovery.

