# Supplementary material for: The effect of sodium-glucose co-transporter-2 (SGLT2) inhibitors on blood interleukin-6 concentration: a systematic review and meta-analysis of randomized controlled trials
Source: BMC Endocr Disord. 2023 Nov 24;23:257. doi: 10.1186/s12902-023-01512-1 (PMC10668472; doi:10.1186/s12902-023-01512-1)
Supplement: Supplementary file 1 — Additional file 1: Table S1. We have searched PubMed, Embase, and Scopus databases using the terms below, PubMed for example (Updated to November 2023). Table S1. PICO inclusion criteria. Table S1. Meta-regression analysis of demographic and clinical variables on IL-6 lowering effect of SGLT2 inhibitors. Figure S1. Forest plot of the effect of SGLT2 inhibitor drugs on interleukin-6 based on the trial duration. Figure S2. Forest plot of the effect of SGLT2 inhibitor drugs on interleukin-6 based on the level of HbA1c. Figure S3. Relationship between level of HbA1c and interleukin-6 lowering effect of SGLT2 inhibitors. SGLT2 inhibitor: Sodium-glucose co-transporter-2 inhibitors. Figure S4. Relationship between male sex and interleukin-6 lowering effect of SGLT2 inhibitors. SGLT2 inhibitor: Sodium-glucose co-transporter-2 inhibitors. Figure S5. Relationship between age and interleukin-6 lowering effect of SGLT2 inhibitors. SGLT2 inhibitor: Sodium-glucose co-transporter-2 inhibitors. Figure S1. Risk of bias quality assessment results using ROB-2 tool. Figure S2. Funnel plot with pseudo 95% confidence limits demonstrating the SMD of interlukin-6 for each trial against their corresponding SEs. SMD: Standardized mean difference, SE: Standard error. Egger regression test had the p-value of 0.061. Figure S3. Galbraith plot to assess heterogeneity of the effects of SGLT2 inhibitor drugs on interleukin-6. SGLT2 inhibitor: Sodium-glucose co-transporter-2 inhibitors. Figure S4. The sensitivity analysis of the included studies using the leave-one-out (Left) and cumulative (Right) approaches. Table S1. Sensitivity analyses to assess the effect of different correlation coefficients on the main results. [file 12902_2023_1512_MOESM1_ESM.pdf]

Supplementary file

Contents

1. Section A 2

Table S1. Detailed search strategy 2

2. Section B 3

Table S1. PICO inclusion criteria. 3

3. Section C 4

Table S1. Meta-regression analysis of demographic and clinical variables on IL-6 lowering effect of SGLT2 inhibitors. 4

Figure S1. Forest plot of the effect of SGLT2 inhibitor drugs on interleukin-6 based on the trial duration. 5

Figure S2. Forest plot of the effect of SGLT2 inhibitor drugs on interleukin-6 based on the level of HbA_{1c}. 6

Figure S3. Relationship between level of HbA_{1c} and interleukin-6 lowering effect of SGLT2 inhibitors 7

Figure S4. Relationship between male sex and interleukin-6 lowering effect of SGLT2 inhibitors 8

Figure S5. Relationship between age and interleukin-6 lowering effect of SGLT2 inhibitors.. 9

4. Section D 10

Figure S1. Risk of bias quality assessment results using ROB-2 tool 10

Figure S2. Funnel plot with pseudo 95% confidence limits. 11

Figure S3. Galbraith plot to assess heterogeneity of the effects of SGLT2 inhibitor drugs on interleukin-6. 12

Figure S4. The sensitivity analysis of the included studies. 13

Table S1. Sensitivity analyses to assess the effect of different correlation coefficients on the main results. 14

1. Section A

Table S1. We have searched PubMed, Embase, and Scopus databases using the terms below, PubMed for example (Updated to November 2023)

Search Number	Query	N
1	"Sodium-Glucose Transporter 2 Inhibitors"[Mesh Terms] OR "Sodium-Glucose Transporter 2 Inhibitors"[Title/Abstract]	6104
2	"Empagliflozin"[Title/Abstract] OR "Canagliflozin"[Title/Abstract] OR "dapagliflozin"[Title/Abstract] OR "ertugliflozin"[Title/Abstract] OR "ipragliflozin"[Title/Abstract] OR "licogliflozin"[Title/Abstract] OR "remogliflozin"[Title/Abstract] OR "sergliflozin"[Title/Abstract] OR "gliflozin"[Title/Abstract]	6088
3	#1 OR #2	9623
4	"Inflammation"[MeSH Terms] OR "Inflammation"[Title/Abstract] OR "inflammat*"[Title/Abstract]	1,446,648
5	"Interleukin-6"[MeSH Terms] OR "Interleukin-6"[Title/Abstract]	109,319
6	"Interleukins"[MeSH Terms] OR "Interleukins"[Title/Abstract]	279,312
7	"Tumor Necrosis Factors"[Title/Abstract] OR "Tumor Necrosis Factors"[MeSH Terms]	172,163
8	"Cytokines"[MeSH Terms] OR "Cytokines"[Title/Abstract] OR "Chemokines"[MeSH Terms] OR "Chemokines"[Title/Abstract]	914,177
9	#4 OR #5 OR #6 OR #7 OR #8	2,030,967
10	"Randomized Controlled Trial"[Publication Type] OR "Controlled Clinical Trial"[Publication Type] OR "Clinical Trial"[Publication Type] OR "Comparative Study"[Publication Type]	2,682,474
11	"Randomized Controlled Trials as Topic"[Mesh Terms] OR "Randomized Controlled Trial"[Title/Abstract] OR "Random Allocation"[Mesh Terms] OR "Random Allocation"[Mesh Terms] OR "random*"[Title/Abstract] OR (("Clin*"[Title/Abstract]) ("Trial*"[Title/Abstract]))	2,012,079
12	"Double Blind Method"[Mesh Terms] OR "Single Blind Method"[Mesh Terms]	208,376

13	((“Singl*”[Title/Abstract] OR “Doubl*”[Title/Abstract] OR “Tripl*”[Title/Abstract]) (“Blind*”[Title/Abstract] OR “Mask*”[Title/Abstract]))	228,871
14	“Placebos”[Mesh Terms] OR “Placebo*”[Title/Abstract]	267,921
15	“Research Design”[Mesh Terms] OR “Follow up Studies”[Mesh Terms] OR “Prospective Studies”[Mesh Terms]	1,711,555
16	“Control*”[Title/Abstract] OR “Prospectiv*”[Title/Abstract] OR “Volunteer*”[Title/Abstract]	5,546,359
17	#10 OR #11 OR #12 OR #13 OR #14 OR #15 OR #16	8,809,038
18	#3 AND #9 AND #17	461

2. Section B

Table S1. PICO inclusion criteria.

PICO	Descriptors
Population	Adult subjects
Intervention	SGLT2 inhibitors
Comparison	Other glycemia-lowering agents, other medications, or placebo
Outcome	Blood IL-6 level

3. Section C

Table S1. Meta-regression analysis of demographic and clinical variables on IL-6 lowering effect of SGLT2 inhibitors.

Variable	**N**	**β**	**95% CI**	**P**
Age	16	0.001	-0.071, 0.073	0.978
HbA1c	10	-0.403	-0.639, -0.166	0.004
Male	15	0.018	-0.017, 0.054	0.280

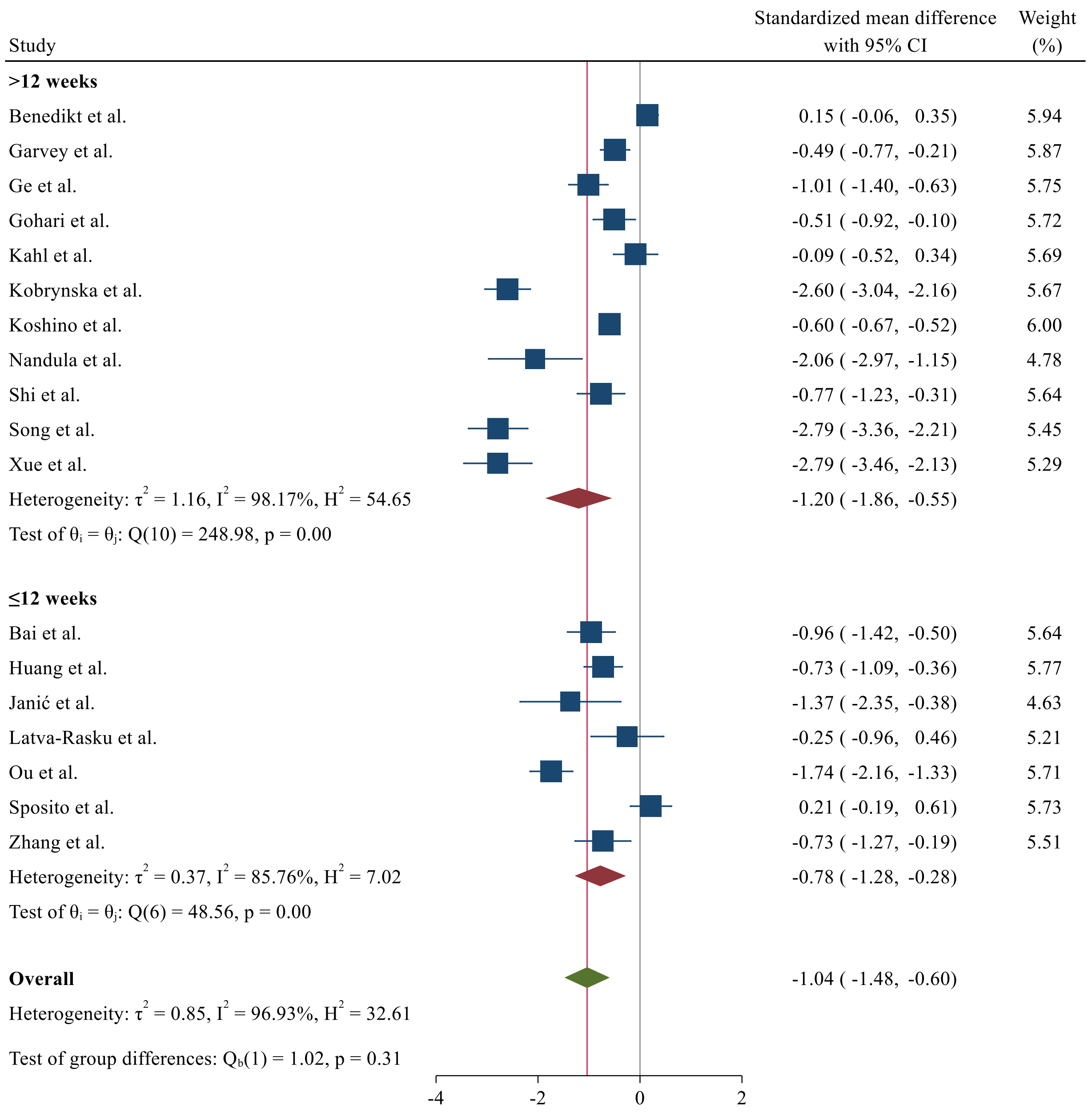

Figure S1. Forest plot of the effect of SGLT2 inhibitor drugs on interleukin-6 based on the trial duration.

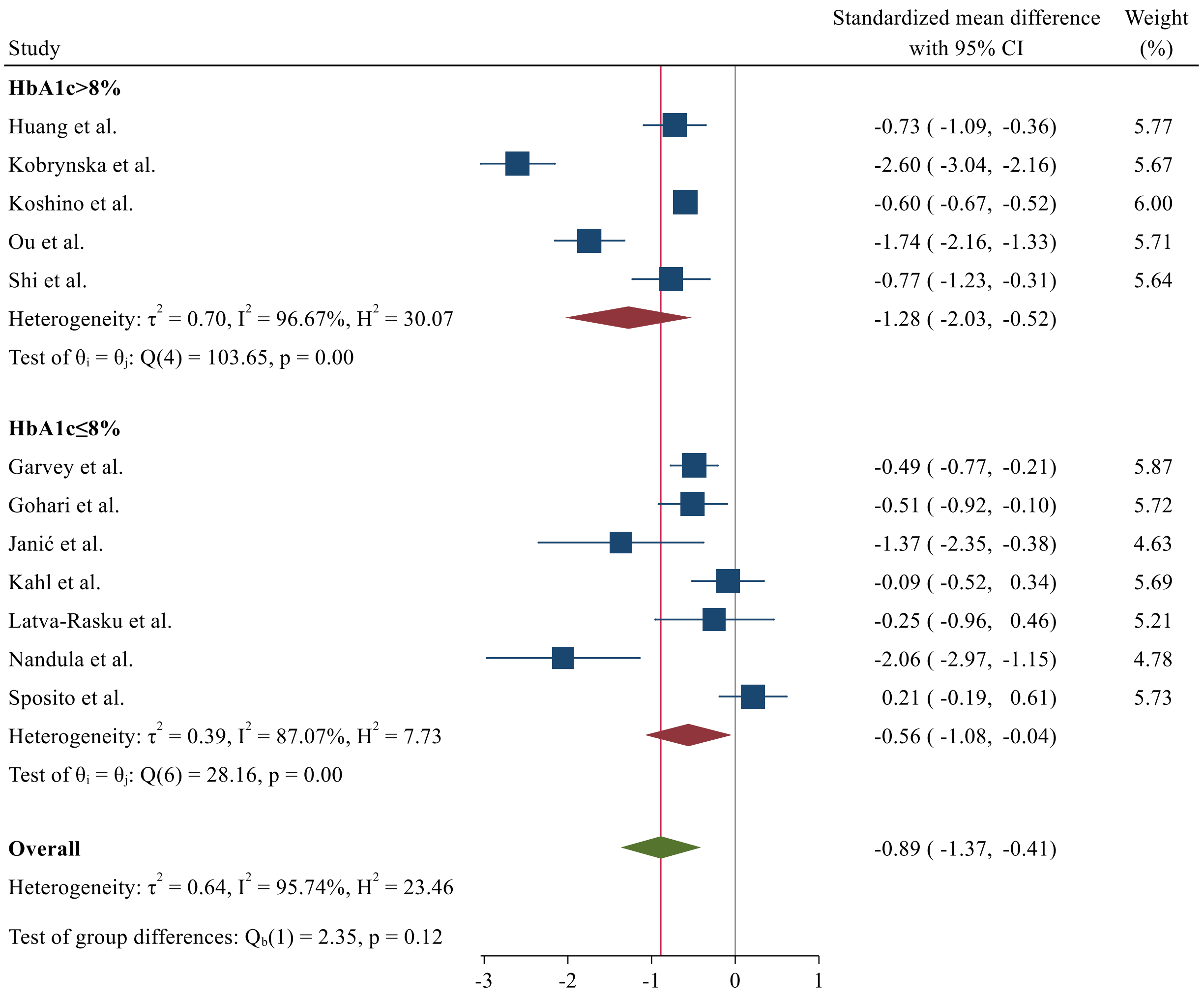

Figure S2. Forest plot of the effect of SGLT2 inhibitor drugs on interleukin-6 based on the level of HbA_{1c}.

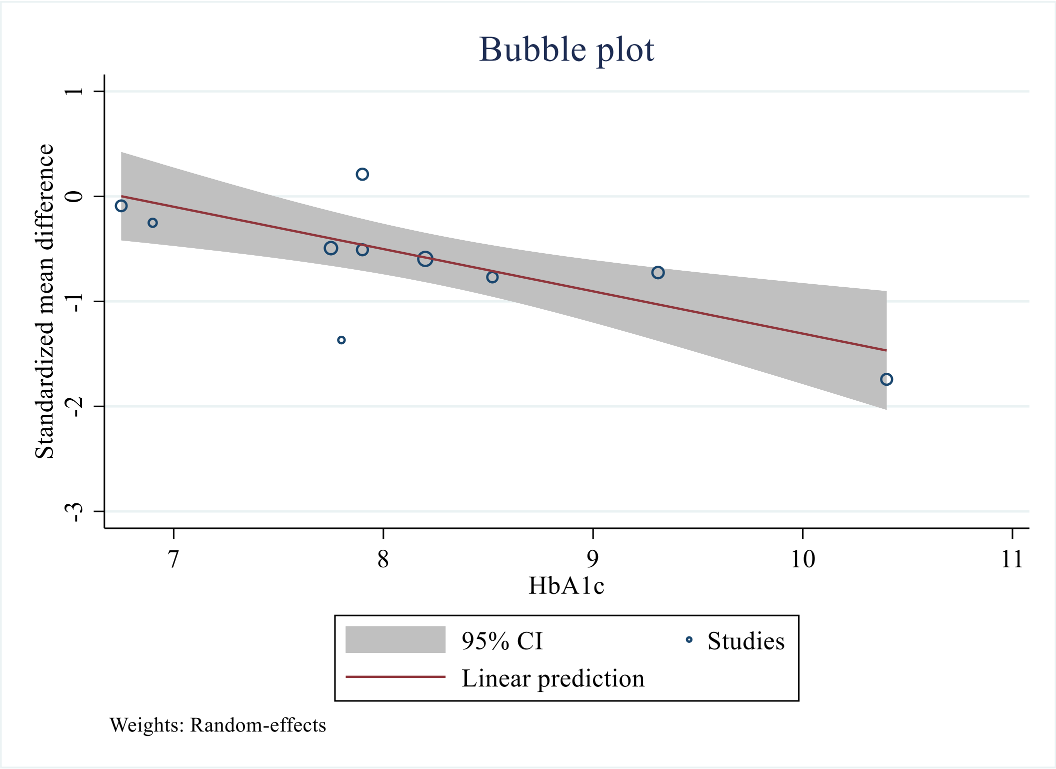

Figure S3. Relationship between level of HbA_{1c} and interleukin-6 lowering effect of SGLT2 inhibitors. SGLT2 inhibitor: Sodium-glucose co-transporter-2 inhibitors.

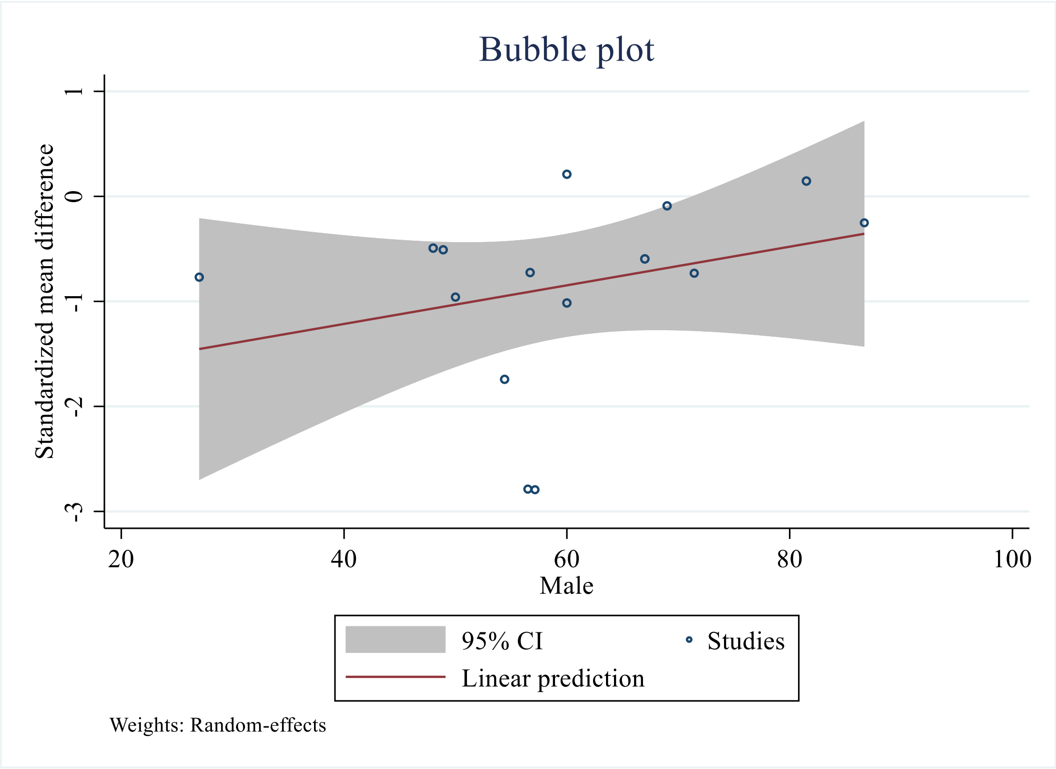

Figure S4. Relationship between male sex and interleukin-6 lowering effect of SGLT2 inhibitors. SGLT2 inhibitor: Sodium-glucose co-transporter-2 inhibitors.

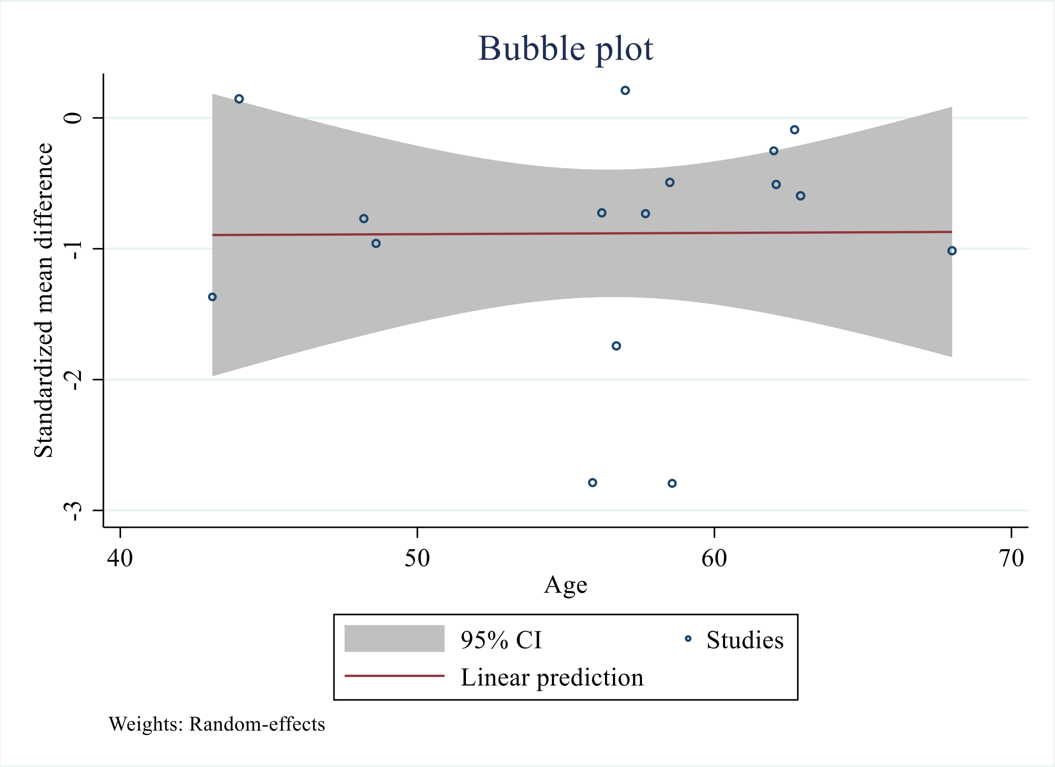

Figure S5. Relationship between age and interleukin-6 lowering effect of SGLT2 inhibitors. SGLT2 inhibitor: Sodium-glucose co-transporter-2 inhibitors.

4. Section D

Unique ID	Experimental	Comparator	Weight	D1	D2	D3	D4	D5	Overall
Shi et al.	Dapagliflozin	Other Antidiabetic Drugs	5.64	-	!	!	+	+	!
Benedikt et al.	Empagliflozin	Placebo	5.94	+	+	+	+	+	+
Song et al.	Dapagliflozin	Anti-heart failure treatment	5.45	-	+	+	!	+	-
Ge et al.	Dapagliflozin	sacubitril+valsartan	6.98	+	!	+	-	+	-
Zhang et al.	Dapagliflozin	placebo	6.63	-	!	+	+	+	-
Ou et al.	Dapagliflozin+Liraglutide	Liraglutide	6.69	-	!	+	!	+	-
Koshino et al.	Canagliflozin	Placebo	7.33	+	+	+	+	!	!
Kobrynska et al.	Empagliflozin	Metformin	6.68	!	!	+	-	+	-
Janić et al.	Empagliflozin	palcebo	5.42	!	+	+	+	+	!
Huang et al.	Dapagliflozin	Valsartan	7	!	!	+	-	+	-
Gohari et al.	Emapgliflozin	Placebo	6.92	+	+	+	+	+	+
Xue et al.	Dapagliflozin	Placebo	6.33	-	!	+	!	+	-
Sposito et al.	Dapagloflozin+metformin	glibnclamide+metformin	6.94	+	+	+	+	+	+
Nandula et al.	Canagliflozin	Placebo	5.63	+	+	+	+	+	+
Kahl et al.	Empagliflozin	Placebo	6.88	+	+	+	+	+	+
Bai et al.	Dapgliflozin+Sitagliptan	Sitagliptan	6.81	-	!	+	-	+	-
Latva-Rasku et al.	Dapagliflozin	Placebo	6.21	+	+	+	+	+	+
Garvey et al.	Canagliflozin	Glimepiride	7.14	+	+	+	+	+	+

+ Low risk
! Some concerns
- High risk

D1 Randomisation process
D2 Deviations from the intended interventions
D3 Missing outcome data
D4 Measurement of the outcome
D5 Selection of the reported result

Figure S1. Risk of bias quality assessment results using ROB-2 tool.

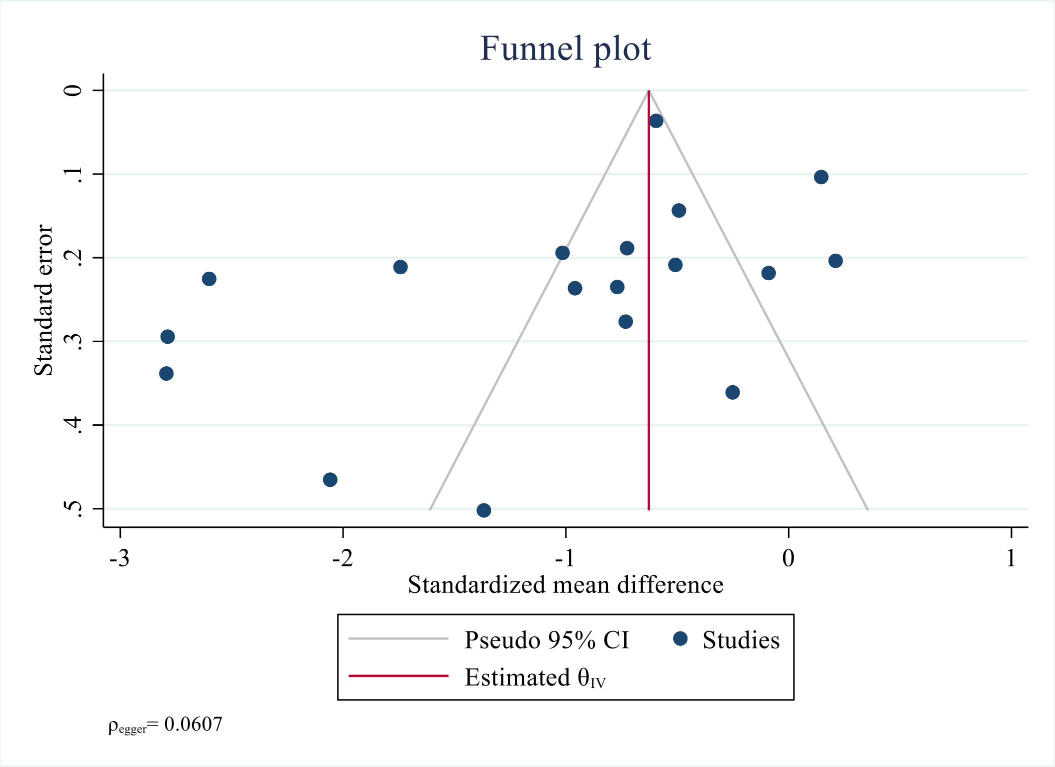

Figure S2. Funnel plot with pseudo 95% confidence limits demonstrating the SMD of interlukin-6 for each trial against their corresponding SEs. SMD: Standardized mean difference, SE: Standard error. Egger regression test had the p-value of 0.061.

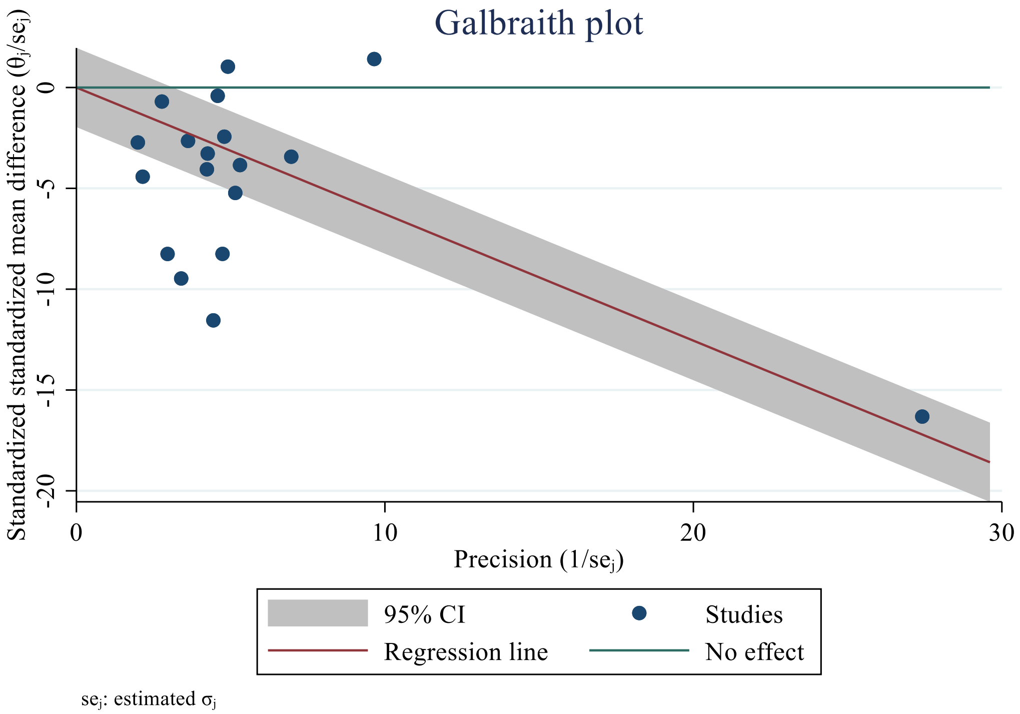

Figure S3. Galbraith plot to assess heterogeneity of the effects of SGLT2 inhibitor drugs on interleukin-6. SGLT2 inhibitor: Sodium-glucose co-transporter-2 inhibitors.

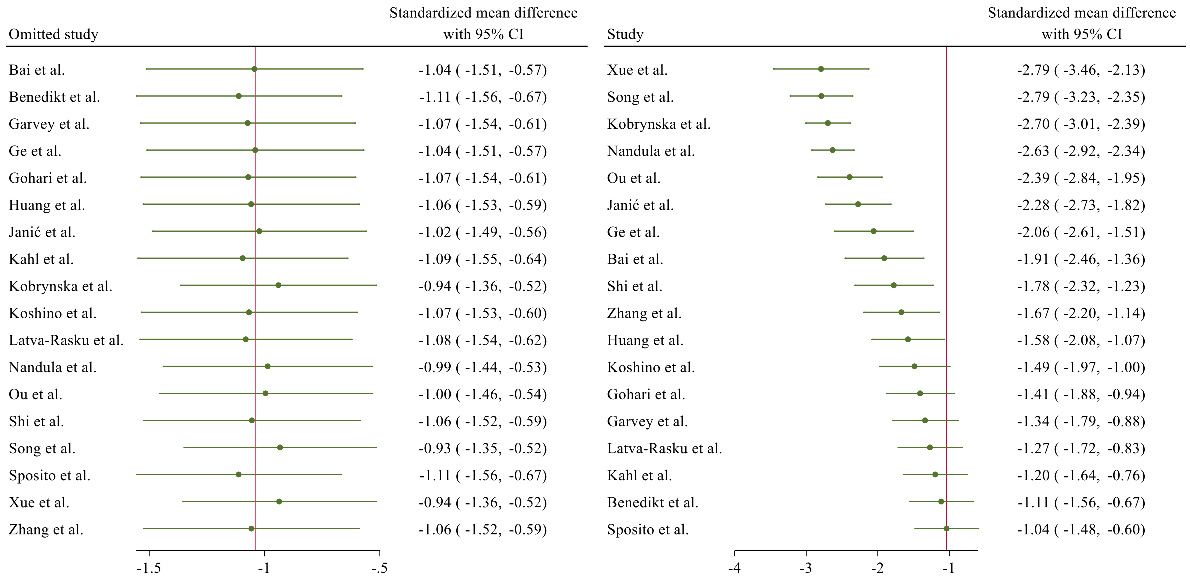

Figure S4. The sensitivity analysis of the included studies using the leave-one-out (Left) and cumulative (Right) approaches.

Table S1. Sensitivity analyses to assess the effect of different correlation coefficients on the main results.

	Correlation Coefficient (r)		
	0.50	0.70	0.90
**MD [95% CI], I^2**	-3.22 [-6.31, -0.14], I^2=99%	-3.31 [-6.51, -0.12], I^2=99%	-3.40 [-6.69, -0.10], I^2=99%
**SMD [95% CI], I^2**	-0.85 [-1.23, -0.47], I^2=95%	-1.04 [-1.51, -0.56], I^2=96%	-1.54 [-2.28, -0.80], I^2=98%

MD: Mean difference, SMD: Standardized mean difference.
